# Supplementary material for: Modeling the relative risk of SARS-CoV-2 infection to inform risk-cost-benefit analyses of activities during the SARS-CoV-2 pandemic
Source: PLoS One. 2021 Jan 28;16(1):e0245381. doi: 10.1371/journal.pone.0245381 (PMC7842882; doi:10.1371/journal.pone.0245381)
Supplement: Attachment — Submitted filename: Review_PONE-D-20-27466.pdf [file pone.0245381.s002.pdf]

Review for publication in PLOS One

'A deterministic linear infection model to inform Risk-Cost-Benefit Analysis of activities during the SARS-CoV-2 pandemic'

(McCarthy, Dumas, McCarthy, and Dewitt)

Summary and Recommendation:

McCarthy et al. have formulated a "deterministic linear" modeling framework to inform risk-cost-benefit analysis of activities during the SARS-CoV-2 pandemic. The model outputs infection probabilities that are additive over defined sub-activities and infection pathways. Namely, the model assumes independence of the individual probabilities and that activities cannot last longer than one day. Then, the model is applied to estimate the risk associated with taking an airplane ride, under varying parameter choices. Furthermore, the authors argue that the linearity assumption makes the model easier to interpret, with minimal sacrifice in reliability.

This is a very timely topic and I would like to applaud the authors for tackling this topic using a more abstract, mathematical framework. The results suggest that the model has a high potential utility in estimating relative risks associated with various activities. I would recommend this paper for acceptance, but believe a couple aspects of the manuscript could be thoroughly revised. First, the paper needs further clarity in terms of definitions (i.e., "deterministic" model), assumptions, and presentation of the mathematics. Second, the model application to the airplane ride example should consider a wider range of parameters and augmented interpretation of estimated risk values. These revisions to the paper will enhance the demonstration of the model's potential ability for application in risk estimation and decision making.

Broader Comments:

I. The model is posited as a "deterministic model" throughout the manuscript. It appears that "deterministic" refers to the fact that the values of the model parameters are deterministic (i.e., the τ parameters in Section 2 and the probability that a neighbor is infected), although this was not explicitly laid out. I believe that more exposition on the meaning of "deterministic" within the context of the model is desirable. In some cases, the usage of the word can be confusing – a few examples below:

   In the Introduction, the authors mention the "advantages of a linear (deterministic) model", as if "linear" and "deterministic" are directly interchangeable. This is somewhat confusing and should be explained more thoroughly!

   The authors differentiate between a "deterministic or statistical" model in Section

2.0.2. What exactly is the difference in this context? After all, we are still concerned with a probabilistic model, which outputs probabilities.

II. The authors applied their model to the specific example of COVID-19 risk on an airplane ride. A brief discussion of how estimated risk differs with varying parameters is provided in Section 3.4-3.5. The number of scenarios considered (three varying airplane distancing and boarding distance, three decay assumptions) is relatively small and should be expanded. Ideally, there should be a visualization that demonstrates how varying certain parameters alters the risk (i.e., figures or counter plots). Some more discussion on the interpretation of the discrepancy in estimated risk values (i.e., 359 for scenario 1 vs. 146 for scenario 2) is also desirable.

Specific comments on manuscript details:

ABSTRACT

- The authors state early on that their model "can produce estimates of relative risk". This is an important point – the distinction between relative and absolute risk, as well as the value of estimating relative risk should be more clearly laid out here.

- "..in which we assume infections come from a series of independent risks." Without having read the entire article, it was not immediately evident to me that "series of independent risks" refers to independence with regards to time interval, mode of transmission, etc. I would consider discussing the independence property earlier in the abstract, and provide some more explanation (of course, the full treatment is still most appropriate for Section 2).

SECTION 2

- Section 2.0.2, A7: In my opinion, this assumption requires more discussion (which was included to some extent in Section 4.1). If a household member is infected, this member presumably has a very high likelihood of infecting other household members upon returning home. This is because household members spend significant time together indoor, share surfaces, and are perhaps rather likely to not wear PPE while at home.

  Ideally, the model would be extended to account for the possibly significant increase in risk from household members doing activities together. If this is not feasible, at least some further discussion may be desirable.

- Section 2.1: Here, a couple examples relating s, p, and π are provided. The authors give a couple estimates of π in the Appendix using real data. I would suggest listing these estimates in Section 2.1, so that the reader has some more context regarding realistic values of π.

- Section 2.2: The model for infection probability by air-borne transmission includes the term $[\text{time of } S_j]$, so it appears that infection probability scales linearly with time spent in the vicinity of an infected neighbor.

  I would suggest adding an assumption (or multiple assumptions) in Section 2.0.2 to differentiate between additive and multiplicative properties for the probabilities. This would account for the linear relationship of the probabilities with $[\text{time of } S_j]$.

- The contention "that this model is strategically valuable even without knowledge of the parameters…" should at least be briefly justified.

<u>SECTION 3</u>

- In the sub-activity decomposition, one sub-activity considered is 'duration of the flight'. I think that activities such as passengers walking around, using the restroom, and interfacing with flight attendants (such as during meal service), etc. need to be accounted for. Or if such activities are insignificant from the risk point-of-view, the insignificance should be justified.

- Sections 3.2-3.3: The calculation of the risk contributions (1.14 in Section 2, 1.84 in Section 3) needs more clarity (either here or in the Appendix). It is not immediately evident from the text how these numbers were obtained.

  Furthermore, the risk in Section 3.2 is calculated to be 27.58. But in Section 3, the authors chose to round this number up to 27.6 It is best to be consistent with significant figures.

- Section 3.5: The authors claim the second decay function "has a very rapid decay" – best to provide a quantitative justification for "very rapid" here.

<u>SECTION 4</u>

- The authors claim that doubling the cleaning frequency will approximately halve the probability that the surface is contaminated. This should be mathematically justified (1-2 sentences is sufficient if the math is trivial).

- It was an excellent idea to include discussion of perceived vs. actual risk. The section could benefit from a few more sentences on how to model and quantify perceived risk, as well as discrepancy between perceived and actual risk. Regarding a decisions-making framework, some more quantitative discussion on how perceived risk could be incorporated is also beneficial.

- Section 4.1: The discussion on limitations is solid but could benefit from more discussion on how much the limitations quantitatively affect the model accuracy, bias, or

estimation confidence. In addition, are these limitations possible to address within the "deterministic linear" framework proposed, or would addressing them require a fundamentally different model?

SECTION 6

- The correlation factor is defined as $f(s)$. Some more clarity regarding why $f(s)$ can be considered the correlation factor is desirable.

- In Section 6.2, the authors thoroughly discuss some estimates of π. However, the link from estimates of π to how close $f(s)$ is to 1 is not entirely evident. Is Table 4, which outputs $f(\pi)$ for each considered value of π, important in this regard? This need to be more clearly discussed.

SECTION 7

- Why omit the proof? Even if the proof is trivial, one should include a few lines sketching how the result is obtained.
