# Supplementary material for: Modeling the relative risk of SARS-CoV-2 infection to inform risk-cost-benefit analyses of activities during the SARS-CoV-2 pandemic
Source: PLoS One. 2021 Jan 28;16(1):e0245381. doi: 10.1371/journal.pone.0245381 (PMC7842882; doi:10.1371/journal.pone.0245381)
Supplement: Attachment — Submitted filename: Review_PONE-D-20-27466R1.pdf [file pone.0245381.s004.pdf]

Review for publication in PLOS One

'Modeling the relative risk of SARS-CoV2 infection to inform Risk-Cost-Benefit Analyses of activities during the SARS-CoV-2 pandemic

(McCarthy, Dewitt, Dumas, and McCarthy)

Summary and Recommendation:

McCarthy et al. have revised their model of SARS-CoV2 relative infection risk, which is used for risk-cost-benefit analyses of typical human activities. Since their first submission of this work to PLOS One, the authors have significantly improved the presentation of their work. The first key change is that the authors have re-posited the model, eliminating reference of the model as "deterministic" and minimizing the ensuing confusion. Second, the authors have significantly expanded the application of their model to human activities. Not only have they improved analysis of the airplane ride case study, but they have also considered several additional environments (stadium, classroom, restaurants, and religious services).

I would recommend publication of this article, and outline some (mostly minor) revisions for the authors to consider. In particular, I would strongly suggest the authors to consider some of my comments regarding modeling and analysis of risk in some of the microenvironments studied (see Section 3).

Specific comments on manuscript details:

<u>ABSTRACT</u>

- A bit more clarity is desired here regarding the distinction between 'activity', 'route of infection', and 'constituent components of activities'.

- I would suggest clarifying what the 'linearity assumption' refers to. This is specified later in the paper, but without further explanation here, it is a bit unclear to the reader.

<u>SECTION 3</u>

- The authors claim that the risk from going to the bathroom is small and thus omit the bathroom analysis from the paper. In my opinion, this merits a brief justification. I would suspect there is nonnegligible risk due to the close distance between two people who are using neighboring urinals.

- Regarding long-range aerosol transmission (i.e., as mentioned in 3.2), the authors may wish to consider investigating the CU Boulder COVID-19 Aerosol Transmission Estimator tool.

- Regarding Section 3.4.1 (Restaurants), I strongly suggest to discuss the risk associated with indoor vs. outdoor dining (a frequently-debated policy issue facing citywide governments). Ideally this should be accompanied by more in-depth discussion on the ratio of aerosol to droplet risk.

- Some further discussion on modeling of trusted cohorts (i.e., in the context of restaurants and religious services) is desirable in the main body of the text.

SECTION 4

- The discussion on household transmission is an important addition. How would the model devised by the authors be applied to household transmission risk? Or would this not be possible? Household transmission has been a large contributor to the late-fall spike in infections within the U.S., so I believe that more discussion on this matter would be desirable.
