# Supplementary material for: Modeling the relative risk of SARS-CoV-2 infection to inform risk-cost-benefit analyses of activities during the SARS-CoV-2 pandemic
Source: PLoS One. 2021 Jan 28;16(1):e0245381. doi: 10.1371/journal.pone.0245381 (PMC7842882; doi:10.1371/journal.pone.0245381)
Supplement: Attachment — Submitted filename: reply to reviewers #2 v1.docx [file pone.0245381.s005.docx]

REVIEWER #1

COVID-19 is a rapidly evolving crisis, and actual data are being gathered on infection sources to help make the type of decisions considered in this paper. Public policy is being driven by accumulating data on infections in restaurants, schools, places of worship etc.. In respect of sports stadiums, a prime concern has been on the travel to stadiums, some of which may involve public transport, and the mingling of fans in congested bars outside the stadiums.

While the analysis that has been undertaken has some merit as an academic exercise, for practical decision-making, this is rather moot. Indeed, with the deployment of effective vaccines in the coming months, the analysis presented will cease to be of much practical relevance by the end of 2021.

**Reply:** Thank you for reading our manuscript and the comments. Given the variation in vaccine distribution both within and between countries – and the international audience of the journal – we believe that jurisdictions exist where our approach could still be helpful for many months to come. There is also the possibility that the current virus will mutate to a point that the vaccine does not work effectively, or a new pandemic could occur with another air-borne virus. As of 12/24/2020, WHO estimates 1.724 million deaths from COVID-19, and in many places there is significant fatigue with broad lockdown measures. A better understanding of which events are more dangerous, and which mitigations strategies are more effective, could significantly help the formulation of public policy.

It could be interesting to adjust the model to account for the percentage of vaccinated persons in the population of potential activity participants (e.g., among sports fans). As far as we know, no population exists yet that is sufficiently vaccinated to produce herd immunity.

REVIEWER #2

Review for publication in PLOS One

*‘Modeling the relative risk of SARS-CoV2 infection to inform Risk-Cost-Benefit Analyses of activities during the SARS-CoV-2 pandemic*

(McCarthy, Dewitt, Dumas, and McCarthy)

**Summary and Recommendation:**

McCarthy et al. have revised their model of SARS-CoV2 relative infection risk, which is used for risk-cost-benefit analyses of typical human activities. Since their first submission of this work to PLOS One, the authors have significantly improved the presentation of their work. The first key change is that the authors have re-posited the model, eliminating reference of the model as “deterministic” and minimizing the ensuing confusion. Second, the authors have significantly expanded the application of their model to human activities. Not only have they improved analysis of the airplane ride case study, but they have also considered several additional environments (stadium, classroom, restaurants, and religious services).

I would recommend publication of this article, and outline some (mostly minor) revisions for the authors to consider. In particular, I would strongly suggest the authors to consider some of my comments regarding modeling and analysis of risk in some of the microenvironments studied (see Section 3).

**Reply:** Thank you for the thorough review of our manuscript and your helpful comments in this and the first review.

ABSTRACT

- A bit more clarity is desired here regarding the distinction between ‘activity’, ‘route of infection’, and ‘constituent components of activities’.
- I would suggest clarifying what the ‘linearity assumption’ refers to. This is specified later in the paper, but without further explanation here, it is a bit unclear to the reader.

**Reply:** We have altered the manuscript to reflect the reviewer’s suggestions.

SECTION 3

- The authors claim that the risk from going to the bathroom is small and thus omit the bathroom analysis from the paper. In my opinion, this merits a brief justification. I would suspect there is nonnegligible risk due to the close distance between two people who are using neighboring urinals.

**Reply:** We added our calculation of the bathroom risk at one stadium. With less mitigation, the risk would be higher; but it is calculable along the same lines as other parts of the event.

- Regarding long-range aerosol transmission (i.e., as mentioned in 3.2), the authors may wish to consider investigating the CU Boulder COVID-19 Aerosol Transmission Estimator tool.

**Reply:** We have included a reference to this model. However, it explicitly excludes calculation of risk from droplets, or any exposure within 6 feet. We know of no published results that allow both droplet and aerosol risk to be included in the same quantitative model, so for now we are using the (admittedly crude) approximation that the aerosol risk is proportionate to the reciprocal of the ventilation per person per unit time. Once data appears that allows both droplet and aerosol risk to be estimated simultaneously, the model should be updated to include these results.

- Regarding Section 3.4.1 (Restaurants), I strongly suggest to discuss the risk associated with indoor vs. outdoor dining (a frequently-debated policy issue facing citywide governments). Ideally this should be accompanied by more in-depth discussion on the ratio of aerosol to droplet risk.

**Reply:** We added some remarks on this – basically being outdoors reduces the aerosol risk dramatically, but not so much the droplet risk. We completely agree that an in-depth discussion on the ratio of aerosol to droplet risk would be incredibly valuable. However, the data does not exist yet.

- Some further discussion on modeling of trusted cohorts (i.e., in the context of restaurants and religious services) is desirable in the main body of the text.

**Reply:** We explained more clearly what we mean by a trusted cohort. If two people are living together with no attempt at self-isolation, then they incur no significant extra risk *from each other* if they attend an event together.

SECTION 4

• The discussion on household transmission is an important addition. How would the model devised by the authors be applied to household transmission risk? Or would this not be possible? Household transmission has been a large contributor to the late-fall spike in infections within the U.S., so I believe that more discussion on this matter would be desirable.

**Reply:** Unfortunately, we believe our model cannot be adapted as it stands to households. Households are not “activities” in our sense of the word, because living in one lasts much longer than a day, by its nature. We also believe that the decomposition of living in a household into subactivities would be much more difficult than the other examples, because of the variation in what might constitute a “subactivity,” and the variation in the ability to restrict subactivities, in the way that a stadium operator might do in order to lower infection risk. We have added more to this section to indicate these limitations of our approach.
